# Low Lung Function Is Associated with Low Baseline Calcaneus Ultrasound T-Score but a Slow Decline in T-Score in a Taiwanese Follow-Up Population with No History of Smoking, Bronchitis, Emphysema, or Asthma

**DOI:** 10.3390/jpm13050795

**Published:** 2023-05-05

**Authors:** Yu-Lin Tsai, Hao-Ping Wang, Da-Wei Wu, Jiun-Chi Huang, Pei-Yu Wu, Szu-Chia Chen

**Affiliations:** 1 Department of Post Baccalaureate Medicine, Kaohsiung Medical University, Kaohsiung 807, Taiwan; 2 Department of Internal Medicine, Kaohsiung Municipal Siaogang Hospital, Kaohsiung Medical University Hospital, Kaohsiung Medical University, Kaohsiung 812, Taiwan; 3 Division of Nephrology, Department of Internal Medicine, Kaohsiung Medical University Hospital, Kaohsiung Medical University, Kaohsiung 807, Taiwan; 4 Division of Pulmonary and Critical Care Medicine, Department of Internal Medicine, Kaohsiung Medical University Hospital, Kaohsiung Medical University, Kaohsiung 807, Taiwan; 5 Faculty of Medicine, College of Medicine, Kaohsiung Medical University, Kaohsiung 807, Taiwan; 6 Research Center for Precision Environmental Medicine, Kaohsiung Medical University, Kaohsiung 807, Taiwan

**Keywords:** lung function, T-score, osteoporosis, Taiwan Biobank, follow-up

## Abstract

Osteoporosis is a common disease, and the prevalence is increasing in patients with chronic respiratory diseases, with important implications with regard to fractures, hospitalization, and death. Due to inconsistent data and a lack of large cohort follow-up studies on the association between lung function and osteoporosis, the aim of this study was to investigate this issue. We enrolled and followed for a median of 4 years a total of 9059 participants with no history of smoking, bronchitis, emphysema, or asthma from the Taiwan Biobank. Spirometry data, including forced expiratory volume in 1 second (FEV1) and forced vital capacity (FVC), were used to assess lung function. Changes in the calcaneus ultrasound T-score (ΔT-score) were calculated as follow-up T-score—baseline T-score. A ΔT-score ≤ −3 (median value of ΔT-score) meant a fast decline in T-score. Multivariable analysis showed that lower values of FEV1 (β, 0.127, *p* < 0.001), FVC (β, 0.203, *p* < 0.001), and FEV1/FVC (β, 0.002, *p* = 0.013) were significantly associated with a low baseline T-score. In addition, after follow-up, higher values of FEV1 (odds ratio (OR), 1.146, *p* = 0.001), FVC (OR, 1.110, *p* = 0.042), and FEV1/FVC (OR, 1.004, *p* = 0.002) were significantly associated with ΔT-score ≤ −3. FEV1/FVC < 70% (OR, 0.838, *p* < 0.001) was significantly associated with ΔT-score ≤ −3. In conclusion, lower FEV1, FVC, and FEV1/FVC were associated with a low baseline T-score, and higher FEV1, FVC, and FEV1/FVC were associated with a rapid decline in T-score in follow-up. This suggests that lung disease may be associated with bone mineral density in the Taiwanese population with no history of smoking, bronchitis, emphysema, or asthma. Further research is needed to establish causality.

## 1. Introduction

Osteoporosis is characterized by low bone mass, disruption of the microarchitecture, and an increase in skeletal fragility [1]. The presence of fragility fractures, particularly of the spine, wrist, hip, humerus, rib, and pelvis, or T-score ≤ −2.5 standard deviations at any site based on bone mineral density (BMD) measurements by dual-energy X-ray absorptiometry (DXA), can be used to make a clinical diagnosis of osteoporosis [2]. Many factors contribute to the risk of osteoporotic fractures, including advanced age, cigarette smoking, prior history of fragility fractures, chronic use of glucocorticoids, low body mass index (BMI), parental history of hip fractures, and excess alcohol intake [3]. The social and economic burden of osteoporosis is steadily increasing because of the aging global population. According to the United States health care system, osteoporotic fractures cost approximately USD 17 billion annually, a figure which is expected to approach USD 50 billion by 2040 [4]. Therefore, it is very important to explore the risk factors for osteoporosis.

Chronic respiratory diseases are those of the airways and other lung structures [5], with common examples including asthma, chronic obstructive pulmonary disease (COPD), pulmonary hypertension, and occupational lung diseases [6]. The World Health Organization (WHO) reported that over 3 million deaths were caused by chronic respiratory diseases in 2019 [6]. In Taiwan, chronic respiratory diseases were the 8th leading cause of death in 2021, with more than 5000 deaths [7]. Smoking tobacco is a known risk factor for chronic respiratory diseases, with others including frequent lower respiratory tract infections during childhood, air pollutants, and occupational chemicals and dust [5]. Previous studies have shown a correlation between chronic lung disease and osteoporosis, including an increase in inflammatory responses such as C-reactive protein and interleukin (IL)-6 [8]. The elevation of inflammation markers has been reported to inhibit bone formation and increase osteoclast activity, thus increasing the risk of osteoporosis and fractures [9]. However, the results of previous studies have been inconsistent, and no large-scale follow-up studies have yet been conducted. Some cell studies in which tissues were collected from the lung have reported that the overexpression of related proteins such as hypoxia-inducible factor (HIF) 1α, vascular endothelial growth factor (VEGF), and VEGF receptor 2 in patients with COPD [10,11], and HIF-1α may stimulate cell growth in a hypoxic environment [12].

There are several studies that pointed out the relationship between COPD and osteoporosis; however, most of them had collected a small number of people, or the follow-up of the studies was short. Due to the inconsistent findings of previous studies and the lack of large-scale cohort follow-up studies on the association between lung function and osteoporosis, the aim of this longitudinal study was to investigate this issue in participants with no history of asthma, smoking, emphysema, or bronchitis. Lung function was evaluated using forced expiratory volume in 1 s (FEV1) and forced vital capacity (FVC), and osteoporosis was evaluated using baseline and change in calcaneus ultrasound T-score (ΔT-score).

## 2. Materials and Methods

### 2.1. Ethics Statement

The Ethics and Governance Council of the TWB and the Institutional Review Board (IRB) on Biomedical Science Research, Academia Sinica, Taiwan, granted ethical approval for the TWB. In addition, the IRB of Kaohsiung Medical University Hospital approved this study (KMUHIRB-E(I)-20210058), which was conducted according to the Declaration of Helsinki.

### 2.2. Taiwan Biobank

The TWB was launched by the Taiwan government in 2012 as a prospective study of citizens 30–70 years of age recruited from centers around Taiwan for research purposes with no history of cancer [13,14]. All enrollees in the TWB sign informed consent forms, after which blood samples are obtained, and in-person interviews with questionnaires and physical examinations are conducted. The Taiwan biobank was initiated and implemented by Academia Sinica (https://www.twbiobank.org.tw) accessed on 8 April 2021. We further requested the data from Academia Sinica and then analyzed it.

Body height, weight, and BMI (kg/m^2^) are recorded during the physical examination. Data collected from the questionnaires include personal information and exercise habits. The “Physical Fitness 333 Plan” criteria were used to define regular exercise as promoted by the Ministry of Education in Taiwan, at least 30 min 3 times a week [15].

**Figure 1:**
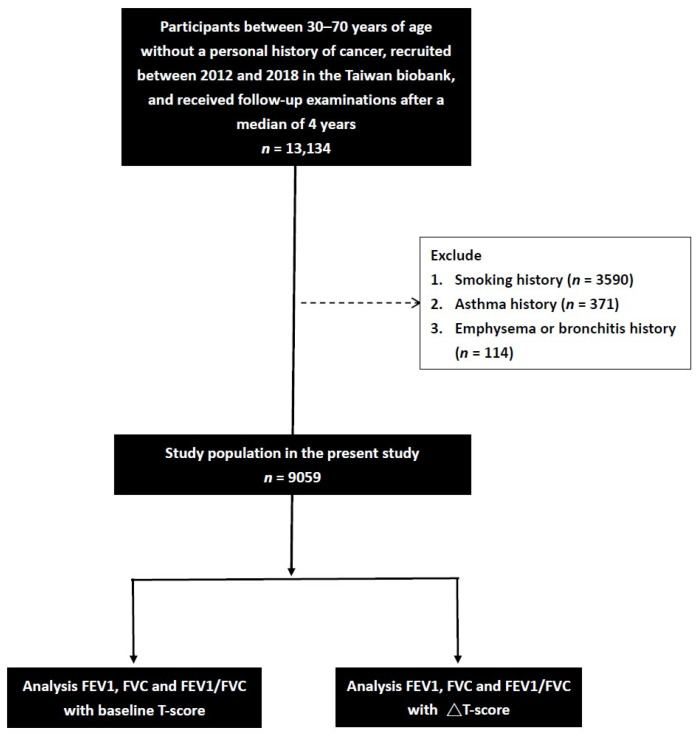
Flowchart of the study population.

We initially identified 13,134 participants with a median of 4 years of complete spirometry data in the TWB. The participants who enroll in the TWB are followed up after 2–4 years. Information, including a questionnaire, physical examination, lung function, calcaneus ultrasound, and blood examination, is collected upon first enrollment and second follow-up. After excluding those with a history of smoking (*n* = 3590), asthma (*n* = 371), and emphysema or bronchitis (*n* = 114), the remaining 9059 participants (1814 males and 7245 females, mean age 51.0 ± 10.2 years) were included (Figure 1).

### 2.3. Laboratory, Medical, and Demographic Variables 

Age, sex, histories of diabetes mellitus (DM) and hypertension, fasting glucose, hemoglobin, triglycerides, total cholesterol, high- and low-density lipoprotein (HDL and LDL) cholesterol, estimated glomerular filtration rate (eGFR using the 4-variable MDRD equation [16]), and uric acid were measured at baseline. Fasting blood samples were obtained from all of the patients, and laboratory tests were conducted using an autoanalyzer (Roche Diagnostics GmbH, D-68298 Mannheim COBAS Integra 400). DM was defined as self-reported fasting glucose level ≥ 126 mg/dL or HbA1c ≥ 6.5%. Participants who had past history of hypertension (self-reported) and whose systolic blood pressure was ≥140 mmHg and diastolic blood pressure was ≥90 mmHg were defined to have hypertension. The data were extracted from TWB (https://www.twbiobank.org.tw) accessed on 8 April 2021.

### 2.4. Assessment of Cigarette Smoking History

All the participants also underwent a face-to-face interview with a researcher, during which they completed a questionnaire asking about cigarette smoking history. Subjects who had smoked one cigarette or more per day for at least 1 year were defined as ever-smokers.

### 2.5. Calcaneus Ultrasound T-Score Measurements

The calcaneus ultrasound T-score (g/cm^2^) in the non-dominant foot was calculated as (T-score of the participant—mean T-score in young adults)/the standard deviation of a normal young-adult population. Changes in the calcaneus ultrasound T-score (ΔT-score) were calculated as follow-up T-score—baseline T-score. ΔT-score ≤ −3 (median value of ΔT-score) meant a fast decline in T-score. All measurements were made using an ultrasound system (Achilles InSight, GE, Madison Heights, Fort Myers, FL, USA).

### 2.6. Spirometry Measurements

Trained technicians performed all FEV1 and FVC measurements using a spirometer with accompanying software (Micro Medical Ltd., Rochester, Kent, UK) following the 2005 European Respiratory Society and American Thoracic Society technical standards [17]. Measurements from the best of three lung function tests were used in the analysis. Reference values were calculated based on a general Asian population and age, sex, and height. Predicted FVC (or predicted FVC%) and predicted FEV1 (or predicted FEV1%) were calculated as the measurements divided by the reference values. Predicted FVC% and FEV1% were calculated using spirometry software. We used the first lung function data to perform further analysis.

### 2.7. Statistical Analysis

Categorical variables are expressed as numbers and percentages, and differences between them were analyzed using the chi-square test. Continuous variables are expressed as mean ± standard deviation, and differences between them were analyzed using the independent t test. Associations among FEV1, FVC, and FEV1/FVC with baseline T-score were examined using multivariable linear regression analysis, and change in T-score ≤ −3 using binary logistic regression analysis. Significant variables in univariable analysis were further put into multivariable analysis. A *p*-value < 0.05 was considered statistically significant. All statistical analyses were performed using SPSS version 19.0 for Windows (IBM Corp., Armonk, NY, USA).

## 3. Results

The enrolled participants (*n* = 9059; 1814/7245 males/females; mean age 51.0 ± 10.2 years) were stratified into 2 groups: those with a baseline calcaneus ultrasound T-score ≥ −2.5 (*n* = 8501; 93.8%) and those with a baseline calcaneus ultrasound T-score < −2.5 (*n* = 558; 6.2%).

### 3.1. Comparisons of Clinical Characteristics between the Baseline T-Score Groups

Compared to the baseline T-score ≥ −2.5 group, the <−2.5 group members were older, had a higher prevalence of hypertension, more regular exercise, lower BMI, higher rate of menopause in females, lower T-score, lower ΔT-score, higher fasting glucose, higher total cholesterol, higher HDL cholesterol and higher LDL cholesterol (Table 1). In addition, the <−2.5 group had lower FEV1 and FVC.

### 3.2. Determinants of Baseline Calcaneus Ultrasound T-Score in Univariable Linear Regression Analysis

Univariable linear regression analysis revealed that older age; male sex; DM; hypertension; lower height; lower BMI; regular exercise; menopause in females; lower eGFR; lower FEV1, FVC, and FEV1/FVC; and higher values of fasting glucose, hemoglobin, triglycerides, total cholesterol, LDL cholesterol, and uric acid were significantly associated with a low baseline T-score (Table 2).

Table 3 shows the associations of FEV1, FVC, and FEV1/FVC with baseline calcaneus ultrasound T-score using multivariable linear regression analysis after adjusting for age, sex, DM, hypertension, height, BMI, regular exercise habits, fasting glucose, hemoglobin, triglycerides, total cholesterol, and LDL cholesterol. eGFR and uric acid (significant variables in Table 2), lower FEV1 (per 1 L; unstandardized coefficient β, 0.127; 95% confidence interval (CI), 0.075 to 0.180; *p* < 0.001), lower FVC (per 1 L; β, 0.203; 95% CI, 0.128 to 0.278; *p* < 0.001), and lower FEV1/FVC (per 1%; β, 0.002; 95% CI, 0 to 0.004; *p* = 0.013) were significantly associated with a low baseline T-score. We further determined the association between FEV1/FVC < 70% and the baseline T-score and found that FEV1/FVC < 70% (β, −0.043; 95% CI, −0.108 to 0.023; *p* = 0.202) was not associated with the baseline T-score.

We have further performed an analysis stratified by sex. In female participants, lower FEV1 (per 1 L; β, 0.181; 95% CI, 0.118 to 0.245; *p* < 0.001), lower FVC (per 1 L; β, 0.291; 95% CI, 0.201 to 0.381; *p* < 0.001), and lower FEV1/FVC (per 1%; β, 0.002; 95% CI, 0 to 0.004; *p* = 0.018) were significantly associated with a low baseline T-score. However, in male participants, FEV1 (*p* = 0.185), FVC (*p* = 0.121), and FEV1/FVC (*p* = 0.478) were not significantly associated with the baseline T-score.

### 3.3. Determinants of Calcaneus Ultrasound ΔT-Score ≤ −3 in Univariable Binary Logistic Regression Analysis

The results of univariable binary logistic regression analysis showed that old age, female sex, lower height, lower BMI, regular exercise, menopause in females, higher total cholesterol, higher HDL cholesterol, lower LDL cholesterol, lower FVC, and higher FEV1/FVC were significantly associated with a ΔT-score ≤ −3 (Table 4).

### 3.4. Associations of FEV1, FVC, and FEV1/FVC with Calcaneus Ultrasound ΔT-Score ≤ −3 in Multivariable Binary Logistic Regression Analysis

After adjusting for age, sex, height, BMI, regular exercise habits, total cholesterol, HDL cholesterol, and LDL cholesterol (significant variables in Table 4), higher FEV1 (per 1 L; odds ratio (OR), 1.146; 95% CI, 1.067 to 1.229; *p* < 0.001), higher FVC (per 1 L; OR, 1.110; 95% CI, 1.004 to 1.228; *p* = 0.042), and higher FEV1/FVC (per 1%; OR, 1.004; 95% CI, 1.002 to 1.006; *p* = 0.001) were significantly associated with a ΔT-score ≤ −3. We further determined the association between FEV1/FVC < 70% and a ΔT-score ≤ −3 and found that FEV1/FVC < 70% (OR, 0.851; 95% CI, 0.780 to 0.930; *p* < 0.001) was significantly associated with a ΔT-score ≤ −3 (Table 5).

We further performed an analysis stratified by sex. In female participants, higher FEV1 (per 1 L; OR, 1.165; 95% CI, 1.069 to 1.270; *p* = 0.001) and higher FEV1/FVC (per 1%; OR, 1.004; 95% CI, 1.001 to 1.007; *p* = 0.002) were significantly associated with a ΔT-score ≤ −3, but FVC was not associated (per 1 L; OR, 1.124; 95% CI, 0.995 to 1.269; *p* = 0.059). However, in male participants, FEV1 (*p* = 0.091), FVC (*p* = 0.430), and FEV1/FVC (*p* = 0.078) were not significantly associated with a ΔT-score ≤ −3.

For evaluation of the effect of participants’ smoking history on the T-score, we further performed analysis after including participants with a smoking history (*n* = 13,134). For baseline T-score, we found similar results that lower FEV1 (per 1 L; β, 0.100; 95% CI, 0.060 to 0.141; *p* < 0.001), lower FVC (per 1 L; β, 0.182; 95% CI, 0.124 to 0.241; *p* < 0.001), and lower FEV1/FVC (per 1%; β, 0.002; 95% CI, 0 to 0.003; *p* = 0.017) were significantly associated with a low baseline T-score. However, for a decrease in T-score, FEV1 (*p* = 0.795), FVC (*p* = 0.709), and FEV1/FVC (*p* = 0.419) were not significantly associated with a ΔT-score ≤ −3 after including participants with a smoking history.

## 4. Discussion

In this study, we investigated the associations among lung function as assessed using FEV1 and FVC with a baseline and changes in their calcaneus ultrasound T-score (ΔT-score). We found that low FEV1, FVC, and FEV1/FVC were associated with a low baseline T-score and that high FEV1, FVC, and FEV1/FVC were associated with a ΔT-score ≤ −3 after follow-up in a Taiwanese population with no history of smoking, bronchitis, emphysema, or asthma.

The finding of an association between lower FEV1, FVC, and FEV1/FVC and a low baseline T-score indicates that lower lung function and osteoporosis are related. Risk factors common to both diseases include smoking [18]; a reduction in physical activity [19,20]; low weight [21]; and disease-specific risk factors such as systemic inflammation [22,23,24,25], Vitamin D deficiency [26,27,28,29], use of glucocorticoids [30,31], anemia [32,33], hypoxemia [34,35], and hypercapnia [36]. However, multilevel interactions between different risk factors have not been well studied [37]. Smoking is one of the most important risk factors for COPD [38], but it is also an independent risk factor for osteoporosis and fragility fractures [19]. Mechanisms of the negative relationship between osteoporosis and smoking are not well understood. The well-known proinflammatory effect of tobacco smoke may affect bone health both directly and indirectly [39]. In addition, nicotine has been reported to potentially directly reduce both osteogenesis and angiogenesis, which play key roles in bone metabolism [18]. Smoking has been associated with altered intestinal permeability and calcium absorption, improving blood acidity, leading to higher bone turnover and alterations in bone collagen synthesis [39]. This can then lead to a vicious cycle, resulting in progressive functional impairment [40]. Physical activity is important for bone health given the role of mechanical stress stimuli on bone tropism and its positive effects on skeletal muscle and bone–muscle crosstalk [19,20]. In addition, many studies have shown an association between low BMI with osteoporosis and fractures in patients with COPD [21]. Most patients with COPD have a low body weight, which can be caused by absorption function, gastrointestinal congestion, hypoxia, reduced appetite, and poor digestion [41]. The decrease in BMD caused by malnutrition may be due to systemic inflammatory responses in COPD patients, such as tumor necrosis factor (TNF)-α. TNF-α is also an effective stimulator of osteoclast bone resorption and an inhibitor of collagen synthesis [42]. The long-term management of COPD frequently requires steroid therapy to improve pulmonary symptoms during both the maintenance phase and exacerbations [31]; however, steroids have been associated with a reduction in BMD and an increased risk of fracture. Glucocorticoids increase bone resorption and reduce bone formation [30], which stimulates the proliferation of osteoclasts through the suppression of osteoprotegerin synthesis, an osteoclast differentiation inhibitor, and induction of a receptor activator of nuclear factor kappa-B (RANK) production, which is necessary for osteoclastogenesis [43]. Elevated levels of glucocorticoids have also been shown to stimulate the synthesis of RANK ligand by pre-osteoblast/stromal cells, supporting net bone resorption and differentiation of osteoclasts [43]. However, data on glucocorticoid medication usage is not recorded in the TWB. This may have influenced the interpretation of the association between lung function and T-scores. Chronic systemic inflammation plays an important role in COPD, and it affects both molecular pathways involved in disease etiology as well as the risk of complications, severity, and functional outcomes [23]. Inflammatory cell-induced cytokines, including matrix metalloproteinases, IL-6, IL-17, osteoprotegerin, and TNF-α, have been strongly associated with the development of osteoporosis [22,23,24,25]. These cytokines are considered to be involved in the pathogenesis of both primary and secondary osteoporosis by regulating the RANK/RANK ligand, leading to osteoporosis [44,45,46]. These mechanisms may explain the relationship between low FEV1, FVC, and FEV1/FVC and low T-scores.

We also found that higher FEV1 and FEV1/FVC were associated with a ΔT-score ≤ −3, representing a fast decline in T-scores. On the other hand, FEV1/FVC < 70% was associated with a ΔT-score ≤ −3, representing a slow decline in T-scores. Reduced oxygen plays a role in pathological conditions, such as ischemia, sleep apnea, COPD, low BMD, and tumor formation, as well as in normal development [47,48]. Hypoxic stimuli, such as low ambient oxygen partial pressure and poor oxygen perfusion and diffusion, may lead to a cellular hypoxic response mainly mediated by HIF transcription factors [49]. Obligate heterodimers, HIFs, are composed of two subunits: a stable β subunit and an O2-labile α subunit [50]. HIF activation results in the stimulation of physiological pathways such as glycolysis, cellular apoptosis, angiogenesis, and pH regulation, all of which play crucial roles in survival in a hypoxic environment [51,52]. The stability of HIF-α is affected by hypoxia through molecular mechanisms, including prolyl hydroxylase domain (PHD) enzymes and von Hippel–Lindau protein. In a well-oxygenated environment, HIF-α is hydroxylated post-translationally by PHDs, followed by interactions with Von Hippel–Lindau tumor suppressor for proteasomal degradation and poly-ubiquitination [53]. However, PHD activity is reduced under hypoxic conditions, leading to the accumulation of HIF-α and translocation to the nucleus. This is followed by the dimerization of HIF-α with HIF-β and binding to the hypoxia response element of HIF target genes [54]. These modifications are mediated by PHDs, whose activities are regulated by O2 availability [55]. PHDs have been proposed to link the cellular O2 concentration to HIF molecular responses. The level of oxygen reaching bone tissue is thought to be around 6.6–8.6%, as measured in bone aspirates [56]. Small changes in inspired O2 or O2 delivery may influence cell homeostasis by stimulating HIF pathways [56,57]. Hypoxia leads to the stabilization of HIFs. HIF-1α induces both osteoclasts and osteoblasts, whereas HIF-2 induces osteoclasts but inhibits osteoblasts. In addition, although both HIF-1/2 can stimulate angiogenesis, only HIF-1 provokes an anabolic response due to the inhibition of osteoblast proliferation and differentiation by HIF-2 [58]. Thus, under hypoxic conditions with the stabilization of both HIF-1 and HIF-2, the anabolic response seems to be limited by HIF-2. However, it is possible that the effects of HIFs may depend on bone cycle and age. While it is clear that HIF-1 plays the most important role with regard to an anabolic response with respect to osteogenesis-angiogenesis, HIF-2 may participate in remodeling by altering key markers of osteoblasts and osteoclasts [59]. Therefore, we hypothesis that high FEV1, FVC, and FEV1/FVC may not be associated with hypoxic stimuli, resulting in a rapid decline in the T-score. Currently, there is a lack of experimental human research investigating the response of bone to hypoxic stimuli, and thus further research is needed.

We further performed an analysis stratified by sex. In female participants, we found similar results in all participants wherein lung function was associated with T-score. However, in male participants, no significant association was noted between lung function and T-score. In addition to the possibility that the number of men was too small, this result may also be due to the fact that men’s smoking causes osteoporosis rather than lung function itself. Therefore, when smoking was excluded, no significant significance could be seen. This issue requires more research to confirm.

In this study, we excluded a history of smoking, asthma, and emphysema or bronchitis, as these are conditions that may influence lung function. However, this makes it difficult to discuss the effect of smoking on osteoporosis in cases mostly involving women. We also tried to further perform analysis after including participants with a smoking history and found similar results—that smoking history, lower FEV1, lower FVC, and lower FEV1/FVC were significantly associated with a low baseline T-score. However, for a decrease in T-score, FEV1, FVC, and FEV1/FVC were not significantly associated with a ΔT-score ≤ −3 after including participants with a smoking history, which may suggest that smoking might influence the association between lung function and T-score change. This issue requires more research to confirm.

The key strengths of this research are the comprehensive follow-up data and large participant cohort. However, there were also some limitations. First, data on medications and other factors that could affect the prevention or development of osteoporosis, such as glucocorticoids, are not recorded in the TWB. This may have influenced the interpretation of the association between lung function and T-score. Moreover, a correlation between chronic lung disease and osteoporosis may be driven by inflammatory responses. However, in the Taiwan Biobank, there was a lack of inflammatory markers, such as C-reactive protein or IL-6. Therefore, we could not validate the correlation. Second, we did not use DXA to confirm the presence of osteoporosis, and used ultrasound instead. Although DXA is the preferred technique for measuring BMD, and the BMD T-score is recommended when developing osteoporosis pharmaceuticals, quantitative ultrasound has the advantages of not requiring exposure to radiation, relatively low cost, and portability. In addition, quantitative ultrasound has been shown to be non-inferior to DXA in identifying osteoporosis in Chinese women [60]. However, Chia-Chi Yen et al. showed there is only a weak correlation between DXA and calcaneal quantitative ultrasonography in the Taiwanese population [61], which may not be well correlated with real bone density. There is a lack of evidence that these correlations have true biologically relevant correlates. The technical issues with the data collection for the biobank include the uncertainty of the quality of the data. This was a statistical analysis of the national data biobank without having control over the adequacy of data collection, the authors’ own clinical confirmation study, or access to a validation cohort. Third, in this study, the population was skewed with only 20% men, which may be due to the fact that we excluded a history of smoking, asthma, and emphysema or bronchitis. This makes it hard to discuss the effect of smoking on osteoporosis and also means that there were more women, which may restrict the generalizability of our findings. Another limitation is that only around 50% of TWB enrollees returned for follow-up assessments, possibly leading to sample bias and affecting our conclusions. In addition, all TWB enrollees were ethnically Chinese, which may restrict the generalizability of our findings to other groups. Lastly, surveys were taken from the volunteer participants, mostly females, which could lead to selection bias and affect the interpretation of confidence intervals and standard errors. Though the majority of the participants were females, these participants were recruited by advertisements posted in apartment complexes and sufficiently represent the Taiwanese population.

## 5. Conclusions

In conclusion, we found that lower FEV1, FVC, and FEV1/FVC were associated with a low baseline T-score, while higher FEV1 and FEV1/FVC were associated with a ΔT-score ≤ −3, indicating a rapid decline in the T-score. This suggests that lung disease may be associated with BMD in the Taiwanese population with no history of smoking, bronchitis, emphysema, or asthma. Further research is needed to establish causality.

## Figures and Tables

**Table 1:** Comparison of clinical characteristics among participants according to T score ≥ −2.5 or <−2.5.

Characteristics	T-Score ≥ −2.5 (*n* = 8501)	T-Score < −2.5 (*n* = 558)	*p*
Age (year)	50.6 ± 10.2	58.2 ± 7.5	<0.001
Male gender (%)	20.0	21.0	0.565
DM (%)	4.3	4.7	0.650
Hypertension (%)	11.1	14.3	0.018
BMI (kg/m^2^)	23.7 ± 3.4	23.1 ± 3.4	<0.001
Regular exercise habit (%)	48.8	58.2	<0.001
Menopause in female (%)	53.5	90.0	<0.001
T-score	−0.16 ± 1.52	−3.09 ± 0.53	<0.001
ΔT-score	−0.35 ± 1.00	0.25 ± 1.31	<0.001
Laboratory parameters			
Fasting glucose (mg/dL)	94.6 ± 17.3	96.5 ± 20.2	0.013
Hemoglobin (g/dL)	13.4 ± 1.5	13.5 ± 1.3	0.097
Triglyceride (mg/dL)	106.2 ± 73.5	102.7 ± 60.6	0.270
Total cholesterol (mg/dL)	195.8 ± 35.2	202.3 ± 34.9	<0.001
HDL cholesterol (mg/dL)	56.0 ± 13.0	57.4 ± 13.8	0.017
LDL cholesterol (mg/dL)	121.3 ± 31.2	124.9 ± 30.0	0.007
eGFR (mL/min/1.73 m^2^)	111.0 ± 25.1	109.8 ± 27.5	0.292
Uric acid (mg/dL)	5.2 ± 1.3	5.2 ± 1.3	0.284
Lung function			
FEV1 (L)	1.95 ± 0.74	1.76 ± 0.71	<0.001
FVC (L)	2.66 ± 0.71	2.41 ± 0.72	<0.001
FEV1/FVC (%)	72.8 ± 18.4	72.7 ± 18.3	0.936

Abbreviations. DM, diabetes mellitus; BMI, body mass index; HDL, high-density lipoprotein; LDL, low-density lipoprotein; eGFR, estimated glomerular filtration rate; FEV1, forced expiratory volume in 1 s; FVC, forced vital capacity.

**Table 2:** Determinants for baseline T-score using univariable linear regression analysis.

Parameters	Baseline T-Score		
	Univariable		
	Unstandardized Coefficient β	95% CI	*p*
Age (per 1 year)	−0.059	−0.062, −0.056	<0.001
Male (vs. female)	−0.262	−0.346, −0.178	<0.001
DM	−0.289	−0.455, −0.1123	0.001
Hypertension	−0.477	−0.583, −0.371	<0.001
Height (per 1 cm)	0.014	0.010, 0.019	<0.001
BMI (per 1 kg/m^2^)	0.031	0.021, 0.041	<0.001
Regular exercise habits	−0.273	−0.340, −0.206	<0.001
Menopause in female	−1.390	−1.461, −1.319	<0.001
Laboratory parameters			
Fasting glucose (per 1 mg/dL)	−0.007	−0.009, −0.005	<0.001
Hemoglobin (per 1 g/dL)	−0.073	−0.096, −0.050	<0.001
Triglyceride (per 1 mg/dL)	−0.001	−0.002, −0.001	<0.001
Total cholesterol (per 1 mg/dL)	−0.004	−0.005, −0.003	<0.001
HDL cholesterol (per 1 mg/dL)	0.001	−0.001, 0.004	0.369
LDL cholesterol (per 1 mg/dL)	−0.003	−0.004, −0.002	<0.001
eGFR (per 1 mL/min/1.73 m^2^)	0.004	0.003, 0.006	<0.001
Uric acid (per 1 mg/dL)	−0.054	−0.080, −0.028	<0.001
Lung function			
FEV1 (per 1 L)	0.288	0.243, 0.333	<0.001
FVC (per 1 L)	0.337	0.290, 0.383	<0.001
FEV1/FVC (per 1%)	0.003	0.002, 0.005	<0.001

Values expressed as unstandardized coefficient β and 95% confidence interval (CI). Abbreviations are the same as in Table 1.

**Table 3:** Relation of FEV1, FVC, and FEV1/FVC to baseline T-score using multivariable linear regression analysis.

Lung Function	Model 1		Model 2		Model 3	
	Unstandardized Coefficient β (95% CI)	*p*	Unstandardized Coefficient β (95% CI)	*p*	Unstandardized Coefficient β (95% CI)	*p*
FEV1 (per L)	0.127 (0.075, 0.180)	<0.001	-	-	-	-
FVC (per 1 L)	-	-	0.203 (0.128, 0.278)	<0.001	-	-
FEV1/FVC (per 1%)	-	-	-	-	0.002 (0, 0.004)	0.013

Values expressed as unstandardized coefficient β and 95% confidence interval (CI). Abbreviations are the same as in Table 1. Adjusted for age, sex, DM, hypertension, height, BMI, regular exercise habits, fasting glucose, hemoglobin, triglyceride, total cholesterol, LDL cholesterol, eGFR, and uric acid (significant variables in Table 2).

**Table 4:** Determinants for ΔT-score ≤ −3 using univariable binary logistic regression analysis.

Parameters	ΔT-Score ≤ −3		
	Univariable		
	Odds Ratio	95% CI	*p*
Age (per 1 year)	1.012	1.007–1.016	<0.001
Male (vs. female)	0.703	0.634–0.780	<0.001
DM	1.143	0.932–1.403	0.199
Hypertension	0.937	0.823–1.068	0.328
Height (per 1 cm)	0.989	0.983–0.994	<0.001
BMI (per 1 kg/m^2^)	0.981	0.969–0.993	0.001
Regular exercise habits	1.097	1.010–1.191	0.028
Menopause in female	1.273	1.160–1.397	<0.001
Laboratory parameters			
Fasting glucose (per 1 mg/dL)	1.001	0.999–1.004	0.217
Hemoglobin (per 1 g/dL)	0.992	0.964–1.020	0.566
Triglyceride (per 1 mg/dL)	1.000	0.999–1.000	0.735
Total cholesterol (per 1 mg/dL)	1.003	1.001–1.004	<0.001
HDL cholesterol (per 1 mg/dL)	1.010	1.007–1.014	<0.001
LDL cholesterol (per 1 mg/dL)	1.001	1.000–1.003	0.036
eGFR (per 1 mL/min/1.73 m^2^)	1.000	0.999–1.002	0.584
Uric acid (per 1 mg/dL)	0.977	0.947–1.008	0.149
Lung function			
FEV1 (per 1 L)	0.960	0.907–1.015	0.149
FVC (per 1 L)	0.871	0.822–0.923	<0.001
FEV1/FVC (per 1%)	1.003	1.001–1.006	0.003

Values expressed as odds ratio and 95% confidence interval (CI). Abbreviations are the same as in Table 1.

**Table 5:** Relation of FEV1, FVC, and FEV1/FVC to ΔT-score ≤ −3 using multivariable binary logistic regression analysis.

Lung Function	Model 1		Model 2		Model 3	
	OR (95% CI)	*p*	OR (95% CI)	*p*	OR (95% CI)	*p*
FEV1 (per 1 L)	1.146 (1.067–1.229)	0.001	-	-	-	-
FVC (per 1 L)	-	-	1.110 (1.004–1.228)	0.042	-	-
FEV1/FVC (per 1%)	-	-	-	-	1.004 (1.002–1.006)	0.001

Values expressed as odds ratio (OR) and 95% confidence interval (CI). Abbreviations are the same as in Table 1. Adjusted for age, sex, height, BMI, regular exercise habits, total cholesterol, HDL cholesterol, and LDL cholesterol (significant variables in Table 4).

## Data Availability

The data underlying this study are from the Taiwan Biobank. Due to restrictions placed on the data by the Personal Information Protection Act of Taiwan, the minimal data set cannot be made publicly available. Data may be available upon request to interested researchers. Please send data requests to Szu-Chia Chen, Division of Nephrology, Department of Internal Medicine, Kaohsiung Medical University Hospital, Kaohsiung Medical University.
